# Long-term salinity is associated with deteriorated soil properties, rhizosphere microbiome restructuring, and elevated wheat crown rot: evidence from a 19-year gradient experiment and a 3-year remediation verification

**DOI:** 10.3389/fpls.2026.1992882

**Published:** 2026-10-23

**Authors:** Yajiao Wang, Sen Han, Xuetong Liu, Hongkai Dang, Chunlian Zheng, Yudong Zheng, Caiyun Cao, Yuxing Wu

**Affiliations:** 1 Hebei Academy of Agriculture and Forestry Sciences/National Collection of Plant-associated Microbes (Hebei)/IPM Innovation Center of Hebei Province/International Science and Technology Joint Research Center on IPM of Hebei Province, Plant Protection Institute, Baoding, China; 2 Dryland Farming Institute of Hebei Academy of Agricultural and Forestry Sciences, Hengshui, China

**Keywords:** rhizosphere microbiome, salinity remediation, soil health, soil salinization, sustainable agriculture, wheat crown rot

## Abstract

Soil salinization threatens global agricultural sustainability and may elevate soil-borne disease risk through coordinated changes in soil conditions and the rhizosphere microbiome, yet long-term field evidence remains limited. Here, a 19-year controlled field experiment with an artificial salinity gradient (non-saline, moderate, and severe) was conducted to characterise salinity-associated responses of soil physicochemical properties, wheat crown rot (WCR), and the rhizosphere microbiome. A 3-year field remediation demonstration was implemented to test whether replacing saline irrigation with non-saline water could reverse soil deterioration and reduce disease severity. The results showed that severe salinity increased soil pH from 7.64 to 8.38 and reduced available phosphorus, total nitrogen, and available potassium by 54.11%, 14.63%, and 37.69%, respectively, while the WCR disease index increased from 10.51 to 32.88. It also reduced microbial richness and diversity, altered rhizosphere community composition, with taxon-specific abundance shifts among halophilic taxa, and redistributed community functional potential, with greater representation of genes associated with compatible-solute metabolism and ion homeostasis alongside changes in carbon, nitrogen, and sulfur metabolism. Remediation reduced soil salinity and pH, improved nutrient availability, and decreased the WCR disease index by 49.17%. Together, our results show that long-term salinization is associated with coordinated deterioration of soil conditions, restructuring of the rhizosphere microbiome, redistribution of microbial functional potential, and increased WCR severity. The remediation experiment showed that reducing salinity improved soil conditions and reduced disease severity. Re-establishing favourable soil abiotic conditions may be a prerequisite for alleviating disease risk in salinized agroecosystems.

## 1 Introduction

Soil salinization is increasingly recognized as a pervasive land-degradation process that threatens crop production and ecosystem functioning, especially across drylands and irrigated agroecosystems (Hassani et al., 2020; Perri et al., 2022; Wang et al., 2025). Climate forcing and hydrological seasonality jointly regulate salt accumulation and leaching efficiency, implying that assessments based solely on aridity may underestimate future salinization risks under changing precipitation regimes (Perri et al., 2022). In parallel, emerging syntheses emphasize that effective salinity mitigation requires combining hydrological engineering, agronomic redesign, and nature-based solutions to restore soil function rather than merely removing salts (Tarolli et al., 2024; Wang et al., 2025).

China faces particularly high pressure from saline–alkali soils, accounting for approximately 10.1% of the world’s saline–alkali land, with long-term challenges in maintaining soil structure, fertility, and sustainable productivity (Lei et al., 2025). Secondary salinization is further promoted by drought intensification, inappropriate irrigation–drainage practices, and intensified inputs, which together accelerate physicochemical deterioration of soils and constrain agricultural sustainability (Hassani et al., 2020; Tarolli et al., 2024; Lei et al., 2025). Beyond direct osmotic and ionic stress on crops, salinization induces systemic soil-health degradation, including structural dispersion and reduced infiltration, nutrient imbalance, and disruption of biological functions that are central to resilient agroecosystems (Hassani et al., 2020; Wang et al., 2025). Such multi-dimensional degradation may indirectly amplify secondary pressures such as soil-borne diseases by reshaping the rhizosphere environment and microbiome assembly (Otlewska et al., 2020), yet the mechanistic links among salinity, soil properties, microbiome shifts, and disease outcomes remain insufficiently substantiated.

The Huang–Huai–Hai Plain, one of China’s major wheat-producing regions, is simultaneously challenged by soil salinization and wheat crown rot (WCR). WCR, primarily caused by Fusarium spp. (notably *Fusarium pseudograminearum*), can cause substantial yield losses and is widely recognized as a major constraint in semi-arid cereal systems (Smiley et al., 2005; Kazan and Gardiner, 2018; Pu et al., 2025; Mapuranga et al., 2026). Disease severity is strongly modulated by environmental stress, with drought commonly exacerbating crown rot impacts; transcriptomic evidence also suggests partially shared regulatory frameworks between drought responses and WCR infection responses in wheat (Su et al., 2024; Pu et al., 2025; Mapuranga et al., 2026). Nutrient management, particularly nitrogen, can further interact with WCR severity and nitrogen-use efficiency, reinforcing the need for integrated disease–fertility strategies (Buster et al., 2023). However, the role of pervasive, long-term salinity stress in governing WCR dynamics has received far less attention than the comparatively well-characterised drought–WCR axis, despite the likelihood that salinity-driven changes in soil structure, pH–ionic balance, and nutrient availability could cascade into altered microbial community assembly and function and, ultimately, disease risk.

At present, saline-soil remediation is often implemented through segmented approaches, including engineering (irrigation–drainage and leaching), agronomic measures, chemical amendments, and biological interventions such as microbial inoculants (Lei et al., 2025). Meanwhile, broader “one health” and plant microbiome frameworks emphasize that soil microbiomes mediate key ecosystem services, including stress tolerance and disease suppression, and that management can shift microbiome assembly with consequences for both plant and ecosystem health (Trivedi et al., 2020; Banerjee and van der Heijden, 2023). Plant-driven recruitment via root exudates is increasingly viewed as a leverage point to guide microbiome assembly and improve plant health outcomes under stress environments (Yang et al., 2025). Together, these advances motivate a more holistic strategy that integrates restoration of soil physicochemical foundations with guided recovery of beneficial microbiomes; however, this integrated approach requires clearer evidence on how salinity, soil properties, rhizosphere microbiome characteristics, and disease severity are associated and on how the soil and disease responses change under practical field management.

To address these gaps, we conducted two complementary field experiments. First, a 19-year controlled field experiment with a defined salinity gradient (non-, moderate-, and severe-salinity) was established to characterise salinity-associated responses of soil physicochemical properties, wheat crown rot (WCR), and the rhizosphere microbiome. Second, a 3-year field remediation experiment was implemented to test whether replacing saline irrigation with non-saline water could reverse soil deterioration and reduce disease severity. The objectives were to: (1) quantify the impact of long-term salinity on soil properties and WCR; (2) characterise associated structural and functional shifts in the bacterial community; (3) characterise the integrative associations among salinity, soil properties, the rhizosphere microbiome, and WCR; and (4) test whether the soil and disease responses are reversible under practical management. This dual design provides a scientific foundation for integrated management strategies that combine physicochemical and microbial remediation for sustainable wheat production in salinized ecosystems.

## 2 Materials and methods

### 2.1 Site description and sample collection

#### 2.1.1 Long-term salinity gradient experiment (2006–2024)

To establish the dose-response relationship of long-term salinity stress on soil ecosystems and crop health under controlled conditions, a long-term salinity gradient field experiment was established in 2006 at the farm of Dryland Farming Institute of Hebei Academy of Agricultural and Forestry Sciences, Hengshui City, Hebei Province, China (37°41′ N, 116°15′ E; 21 m a.s.l.). It has a subtropical monsoon climate, with a mean annual temperature of approximately 12.6 °C and a mean annual precipitation of approximately 522 mm. Winter wheat is rotated with summer maize.

The experiment had three treatments: (1) Non-Sal, irrigated with clean water without adding salt; (2) Mid-Sal, irrigated with 0.4% salt water; (3) Hea-Sal, irrigated with 0.8% salt water. Treatment allocation was randomized within blocks, with three independent 57 m^2^ (6 × 9.5 m) plots per treatment; each plot received its assigned irrigation treatment throughout the experiment. The Non-Sal treatment was irrigated with local deep groundwater without additional salt. Saline irrigation water was prepared by mixing sea salt with the same deep groundwater in a 14-m³ mixing pool. The prepared water was pumped through pipelines to the corresponding plots. Winter wheat was generally irrigated two to three times during each growing season. Pre-sowing irrigation was applied when the relative soil water content in the 0–20 cm layer was below 70%; otherwise, it was omitted. Subsequent irrigations were applied at the jointing and flowering stages. Each irrigation event supplied 60 mm of water (600 m³ ha^-^¹), equivalent to 3.42 m³ for each 57-m² plot. Irrigation timing and volume were kept identical among salinity treatments, with irrigation-water salinity being the only difference among treatments. The same treatment was applied to each plot for 19 years.

Rhizosphere soil samples were collected on May 15, 2024 (wheat filling stage). The soil loosely attached to the root surface was first removed by gentle manual shaking. Roots with tightly attached soil were then placed in sterilized water and shaken at 200 rpm for 30 min, finally, the rhizosphere soil was collected by centrifugation at 12, 000 g for 5 min (Edwards et al., 2015). A portion of the collected soil sample was stored at -80 °C for DNA extraction, and the remaining portion was air-dried under ambient temperature and stored at 4 °C for soil analysis.

#### 2.1.2 Field remediation experiment (2022–2025)

To test the management responsiveness and reversibility of salinity-associated soil deterioration and WCR severity identified the long-term controlled experiment in a real and complex field environment, we conducted a 3-year remediation verification experiment from 2022 to 2025 in a typical naturally salinized wheat field in Handan City, Hebei Province (36°36′ N, 114°29′ E), which has the same climate and soil type as the long-term gradient experiment site. The site had been irrigated with local saline groundwater (mineralization degree ~0.55%) for over a decade, resulting in a stable secondary salinization soil and perennial severe incidence of wheat crown rot. This made it an ideal “natural validation site” for studying the co-occurrence of salinization and soil-borne disease and for testing the efficacy of remediation measures.

Two treatments were set up: (1) Remediation group (Rem-Sal): converted to non-saline water irrigation in 2022, with irrigation frequency and amount consistent with the long-term gradient experiment; (2) Control group (Con-Sal): continued irrigation with the original saline groundwater (0.55%), with the same irrigation management as the Rem-Sal group. Each treatment had three replicates, and the plot area was 40 m² (5 × 8 m), arranged in a random block design.

Rhizosphere soil samples and wheat plants were collected on May 15, 2025 (wheat filling stage), following the same sampling method as the long-term gradient experiment. All collected soil samples were air-dried at room temperature and stored at 4 °Cfor soil physicochemical property analysis.

### 2.2 Evaluation of the occurrence of wheat crown rot

Wheat samples were collected on May 15, 2024 (wheat filling stage) according to the “W” multipoint sampling method, 10 sampling points were sampled in each plot, and 10 wheat plants were collected at each sampling point. The severity of wheat crown rot was scored on a 0 to 4 scale based on percentage of crown area with necrosis lesions (Wildermuth and McNamara, 1994). Level 0: completely healthy; level 1: less than 25% necrosis; level 2: 25-50% necrosis; level 3: 51-75% necrosis; level 4: greater than 75% necrosis. The disease index was then calculated as ∑ (Number of diseased plants with each score × score)/(Total number of plants × Highest score) × 100%.

### 2.3 Soil chemical property analysis

Air-dried soil samples were ground and passed through a 2 mm sieve before analysis. Soil organic carbon (SOC) was determined by dichromate oxidation with external heating followed by titration/spectrophotometric endpoint, consistent with improved chromic-acid oxidation procedures (Bowman, 1998; Heanes, 1984). Soil total nitrogen (TN) was determined using Kjeldahl digestion followed by distillation–titration (Nelson and Sommers, 1980). Soil available phosphorus (AP) was extracted using 0.5 M NaHCO_3_ (Olsen-type bicarbonate extraction) and quantified by the molybdenum blue method (Murphy and Riley, 1962). Soil available potassium (AK) was extracted using neutral 1 M ammonium acetate (NH_4_OAc, pH 7.0) and determined by flame photometry/atomic spectrometric measurement as described for NH_4_OAc-extractable K (Heinen Brown et al., 1999). Soil water-soluble salt content (SSC) was determined gravimetrically by evaporating a known volume of soil–water extract to dryness at 105 °C and calculating residue mass per unit soil mass (Hardie and Doyle, 2012; Jia et al., 2025).

### 2.4 DNA extraction, library construction, and metagenomic sequencing

Microbial genomic DNA was extracted from 0.5 g rhizosphere soil with the E.Z.N.A. Soil DNA Kit (Omega Bio-TEK, Norcross, GA, USA) according to the manufacturer’s instructions. TBS-380 Mini-Fluorometer (Turner Biosystems, CA, USA) and NanoDrop 2000 UV-Vis spectrophotometer (Thermo Scientific, Wilmington, DE, USA) were used to determine the concentration and purity of extracted DNA. DNA quality was determined by 1% agarose gel electrophoresis. Covaris M220 (Gene Company Limited, China) was used to fragment DNA to an average size of ~400 bp to construct paired-end libraries. Paired-end libraries were prepared using the NEXTFLEX Rapid DNA-Seq kit (Bioo Scientific, Austin, TX, USA) and sequenced on an Illumina NovaSeq platform. Sequence data associated with this project have been deposited in the NCBI Short Read Archive database (Accession Number: PRJNA1414827).

### 2.5 Sequence assembly, taxonomy and functional annotation

Paired-end Illumina reads were quality-trimmed and adapters removed using fastp (v0.20.0) (Chen et al., 2018). Metagenomic assembly was performed using MEGAHIT (v1.1.2) (Li et al., 2015). Contigs ≥300 bp were retained for downstream gene prediction and annotation. Representative non-redundant gene catalog sequences were aligned to the NCBI NR database using DIAMOND (v0.8.35) with an e-value cutoff of 1e^−5^ for taxonomic annotation (Buchfink et al., 2015). KEGG functional annotation was conducted using DIAMOND against the KEGG database with an e-value cutoff of 1e^−5^ (Kanehisa et al., 2021).

### 2.6 Statistical analyses

Shannon and Chao indices were calculated using Mothur version v.1.30, (http://www.mothur.org/) and the vegan package in R. All univariate response variables, including soil physicochemical properties, alpha-diversity indices, and the disease index, were first tested for normality using the Shapiro–Wilk test and for homogeneity of variances using Levene’s test. Variables satisfying both assumptions were compared among treatments by one-way analysis of variance (ANOVA) followed by Tukey’s *post hoc* test; variables violating either assumption were compared using the nonparametric Kruskal–Wallis test followed by Dunn’s *post hoc* test, with P values adjusted by the Benjamini–Hochberg (BH) false discovery rate (FDR) procedure. For taxonomic analyses, the relative abundances of all genera were tested individually among treatments, and P values were corrected for multiple testing using the BH–FDR procedure, with the number of tests (m) equal to the total number of genera tested. For KO-level functional analyses, KOs were grouped into two predefined functional gene sets: (i) genes involved in carbon, nitrogen, sulfur, and phosphorus metabolism and (ii) genes involved in salt-adaptation traits. Within each gene set, individual KOs were tested for differential abundance among treatments, and P values were corrected within the gene set using the Benjamini–Hochberg (BH) false discovery rate (FDR) procedure, with the number of tests equal to the number of KOs in that gene set. Adjusted P values (q) < 0.05 were considered statistically significant, whereas FDR-adjusted P values between 0.05 and 0.10 were interpreted as marginal evidence and are reported as such. Unless otherwise stated, statistical significance was defined as P < 0.05. Data are presented as mean ± standard deviation (SD). All univariate statistical analyses were conducted in SPSS version 16.0, with Dunn’s *post hoc* tests performed in R; taxonomic, functional, and multivariate analyses were conducted in R.

## 3 Results

### 3.1 Long-term salinity was associated with altered soil physicochemical properties and increased WCR severity

Long-term saline-water irrigation was associated with a clear, salinity-dependent deterioration of rhizosphere soil properties (**Table 1**). Soil soluble salt content (SSC) increased from 0.88 ± 0.02 g kg^-^¹ in Non-Sal to 2.93 ± 0.05 and 4.62 ± 0.07 g kg^-^¹ in Mid-Sal and Hea-Sal, respectively, corresponding to 3.3- and 5.2-fold increases. Soil pH also increased progressively from 7.64 in Non-Sal to 7.80 in Mid-Sal and 8.38 in Hea-Sal. In parallel, soil nutrient availability declined with increasing salinity. Relative to Non-Sal, SOC, TN, AP, and AK decreased by 5.07%, 2.38%, 8.19%, and 34.19%, respectively, in Mid-Sal, and by 5.48%, 14.63%, 54.11%, and 37.69% in Hea-Sal (**Table 1**). Among these variables, AP showed the largest decline under severe salinity.

**Table 1:** Means ± standard deviation of soil chemical properties and of wheat crown rot disease index.

Treatments	Treatments	SSC	SOC	TN	AP	AK	pH	WCR
		g/kg	g/kg	g/kg	mg/kg	mg/kg		
Long-term gradient experiment	Non-Sal	0.88 ± 0.02e	44.34 ± 0.19 a	2.94 ± 0.04 a	39.33 ± 0.25 a	1245.65 ± 23.64 a	7.64 ± 0.04 d	10.51 ± 1.05 d
	Mid-Sal	2.93 ± 0.05c	42.09 ± 0.09 b	2.87 ± 0.07 a	36.11 ± 0.13 b	819.84 ± 20.83 c	7.80 ± 0.04 c	28.1 ± 1.58 c
	Hea-Sal	4.62 ± 0.07a	41.91 ± 0.12 b	2.51 ± 0.04 b	18.05 ± 0.42 d	776.06 ± 12.55 c	8.38 ± 0.03a	32.88 ± 2.36 b
Field remediation experiment	Con-Sal	3.28 ± 0.05b	41.98 ± 0.11 b	2.69 ± 0.05 b	27.53 ± 0.28 c	798.25 ± 15.64 c	8.05 ± 0.04 b	53.24 ± 3.25 a
	Rem-Sal	1.39 ± 0.03d	42.58 ± 0.13 b	2.78 ± 0.04 a	35.26 ± 0.32 b	984.25 ± 15.36 b	7.79 ± 0.04 c	27.06 ± 1.89 c

Different lowercase letters within a column indicate significant differences among all treatments (p < 0.05) according to one-way ANOVA (long-term gradient experiment) or independent samples t-test (field remediation experiment) followed by Tukey’s test. Non-Sal, Non-saline irrigation (clean water); Mid-Sal, Moderate salinity irrigation (0.4% salt water); Hea-Sal, Severe salinity irrigation (0.8% salt water); Con-Sal, Continuous saline groundwater irrigation; Rem-Sal, Converted to non-saline groundwater irrigation. SSC, Soil salt content; SOC, Soil organic carbon; TN, Total nitrogen; AP, Available phosphorus; AK, Available potassium; WCR, disease index of wheat crown rot.

Wheat crown rot (WCR) severity increased concurrently with soil salinization (**Figure 1**; **Table 1**; **Supplementary Table S1**). The disease index increased from 10.51 in Non-Sal to 28.10 in Mid-Sal and 32.88 in Hea-Sal, representing increases of 167.4% and 212.8%, respectively. Thus, increasing salinity was accompanied by progressive soil alkalization and nutrient depletion together with a marked increase in WCR severity.

**Figure 1:**
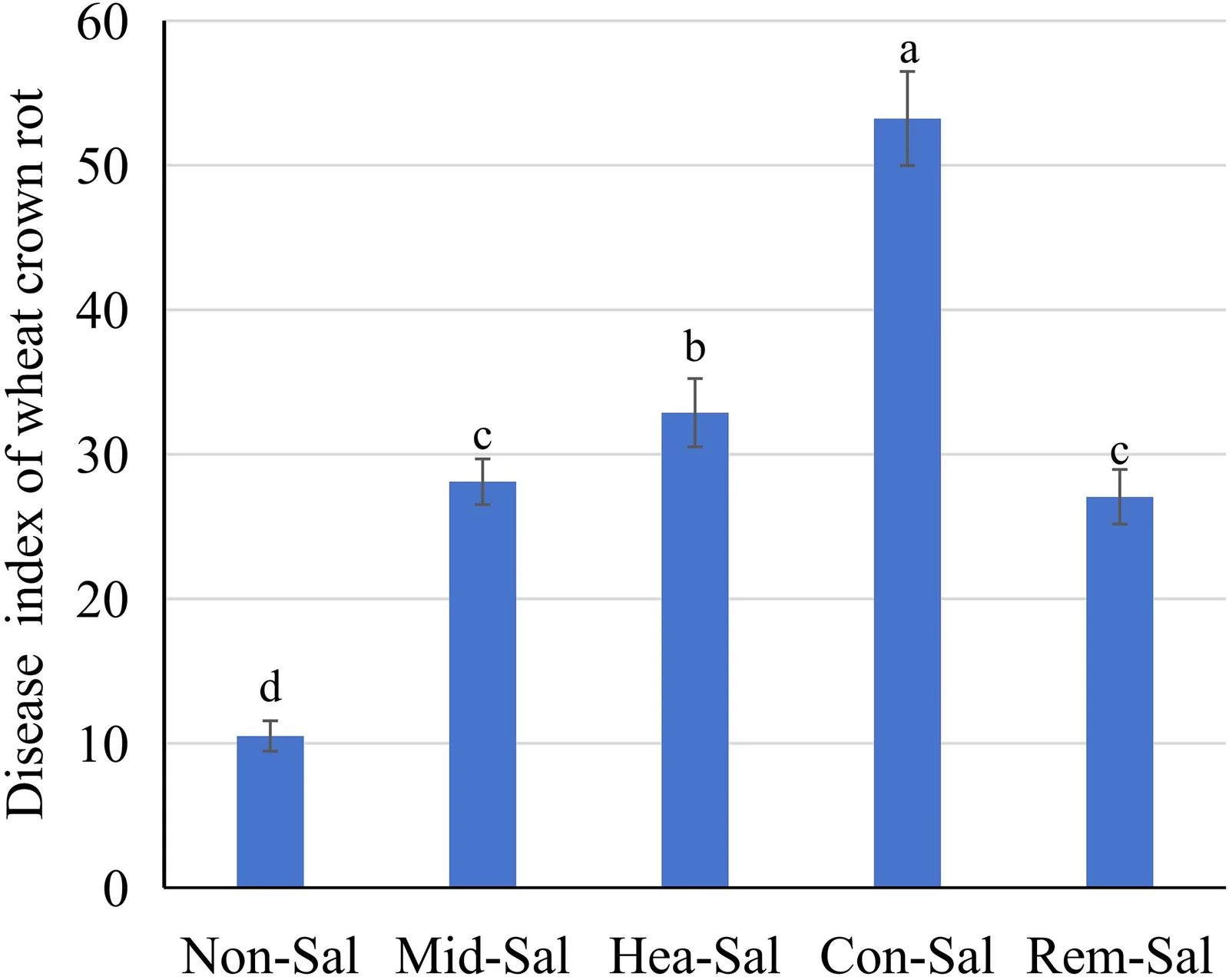
Investigation of wheat crown rot. Bars labelled with different letters differ significantly (p < 0.05) according to Tukey’s test. Non-Sal, Non-saline irrigation (clean water); Mid-Sal, Moderate salinity irrigation (0.4% salt water); Hea-Sal, Severe salinity irrigation (0.8% salt water); Con-Sal, Continuous saline groundwater irrigation; Rem-Sal, Converted to non-saline groundwater irrigation.

### 3.2 Effects of salinity stress on the diversity of soil microbial communities in the wheat rhizosphere

Metagenomic sequencing yielded 45074338, 53556529, 45711094 clean reads and 154693, 203032, 281965 contigs for Non-Sal, Mid-Sal and Hea-Sal respectively. In total, 247 phyla, 5115 genera and 39636 microbial species were identified after taxonomic annotation. To evaluate the effects of salinity stress on soil microbial richness and diversity, indices of Chao and Shannon were calculated respectively (**Figures 2A, B**). The Chao and Shannon indices of Mid-Sal were 12501 and 6.88 respectively, slightly lower than those of Non-Sal (by 0.27% and 0.33%, respectively), but the differences were not significant. The Chao and Shannon indices of Hea-Sal were 12, 122 and 6.72, respectively, which were significantly lower than those of Non-Sal; however, the reductions were modest in magnitude (3.29% and 2.61%, respectively). These results indicate that heavy salinity stress significantly but modestly reduced soil microbial diversity, whereas moderate salinity stress had no significant effect.

**Figure 2:**
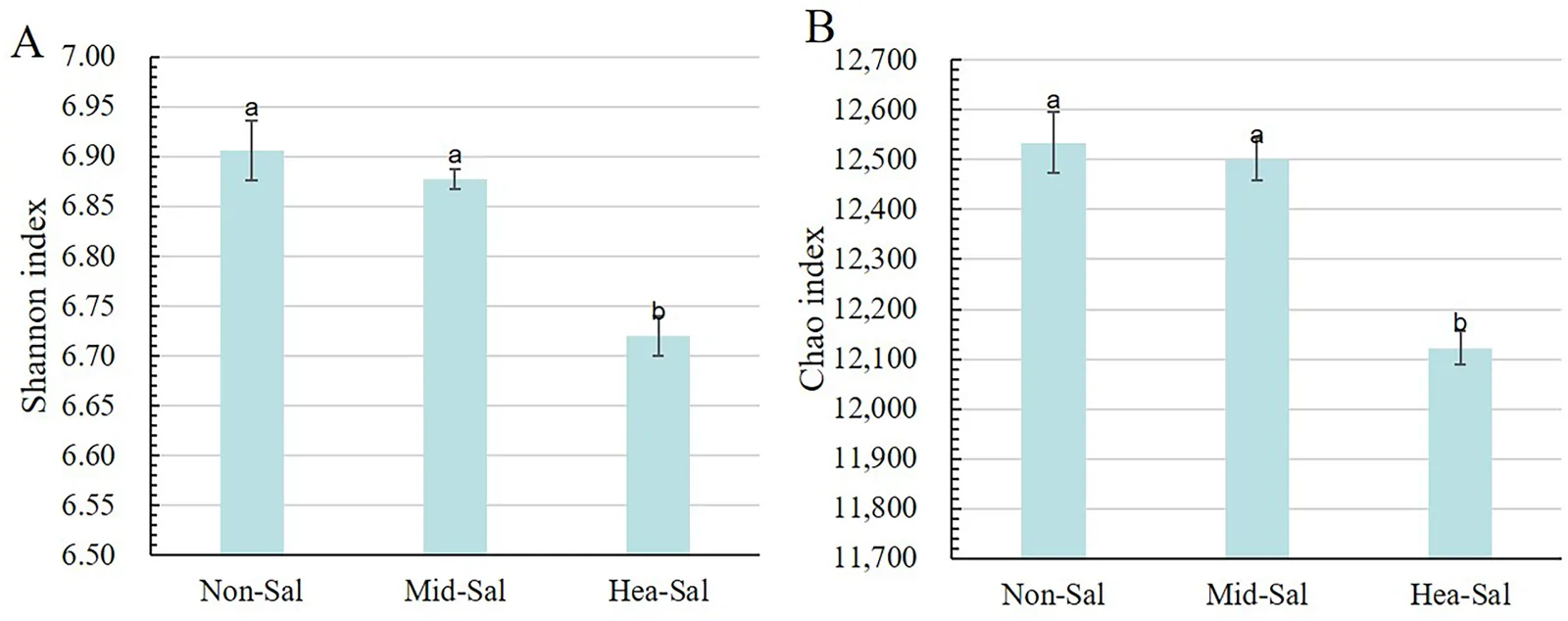
Alpha diversity of the wheat rhizosphere microbial community under different salinity treatments. Shannon diversity index **(A)** and Chao richness index **(B)** of the rhizosphere microbial community. Different lowercase letters above the bars indicate significant differences among salinity treatments (P < 0.05). Non-Sal, non-salinity treatment; Mid-Sal, moderate-salinity treatment; Hea-Sal, heavy-salinity treatment.

### 3.3 Effects of salinity stress on the composition of the soil microbial community in wheat rhizosphere

Taxonomic annotation indicated that 89.57% of sequences were Bacteria, with 1.19%, 0.25%, and 0.028% were assigned to Archaea, Eukaryota, and viruses, respectively. There were 18, 20, and 22 dominant genera (abundance > 1%) in Non-Sal, Mid-Sal, and Hea-Sal, respectively (**Figure 3A**; **Supplementary Table S2**). Twelve genera were consistently dominant across all three treatments, including *Nocardioides*, *Luteimonas*, *Devosia*, *Pseudomonas*, *Croceibacterium*, *Microbacterium*, *Lysobacter*, *Solirubrobacter*, *Sphingopyxis*, *Brevundimonas*, *Sphingomonas*, and *Mesorhizobium*. In Non-Sal, the most abundant genera were *Luteimonas* (7.48%), *Nocardioides* (5.64%), *Pseudomonas* (5.29%), *Devosia* (4.85%), *Lysobacter* (4.20%), and *Pseudoxanthomonas* (3.57%). Under Mid-Sal, *Nocardioides* (7.57%) and *Devosia* (7.46%) became the two most abundant genera, followed by *Luteimonas* (5.03%), *Microbacterium* (3.80%), and *Pseudomonas* (3.68%). Under Hea-Sal, *Nocardioides* further increased to 9.80% and became the predominant genus, whereas *Luteimonas*, *Devosia*, *Croceibacterium*, *Microbacterium*, and *Pseudomonas* accounted for 4.49%, 3.67%, 3.62%, 2.52%, and 2.18%, respectively. These results showed that increasing salinity substantially altered the relative contributions of dominant genera in the wheat rhizosphere microbial community.

**Figure 3:**
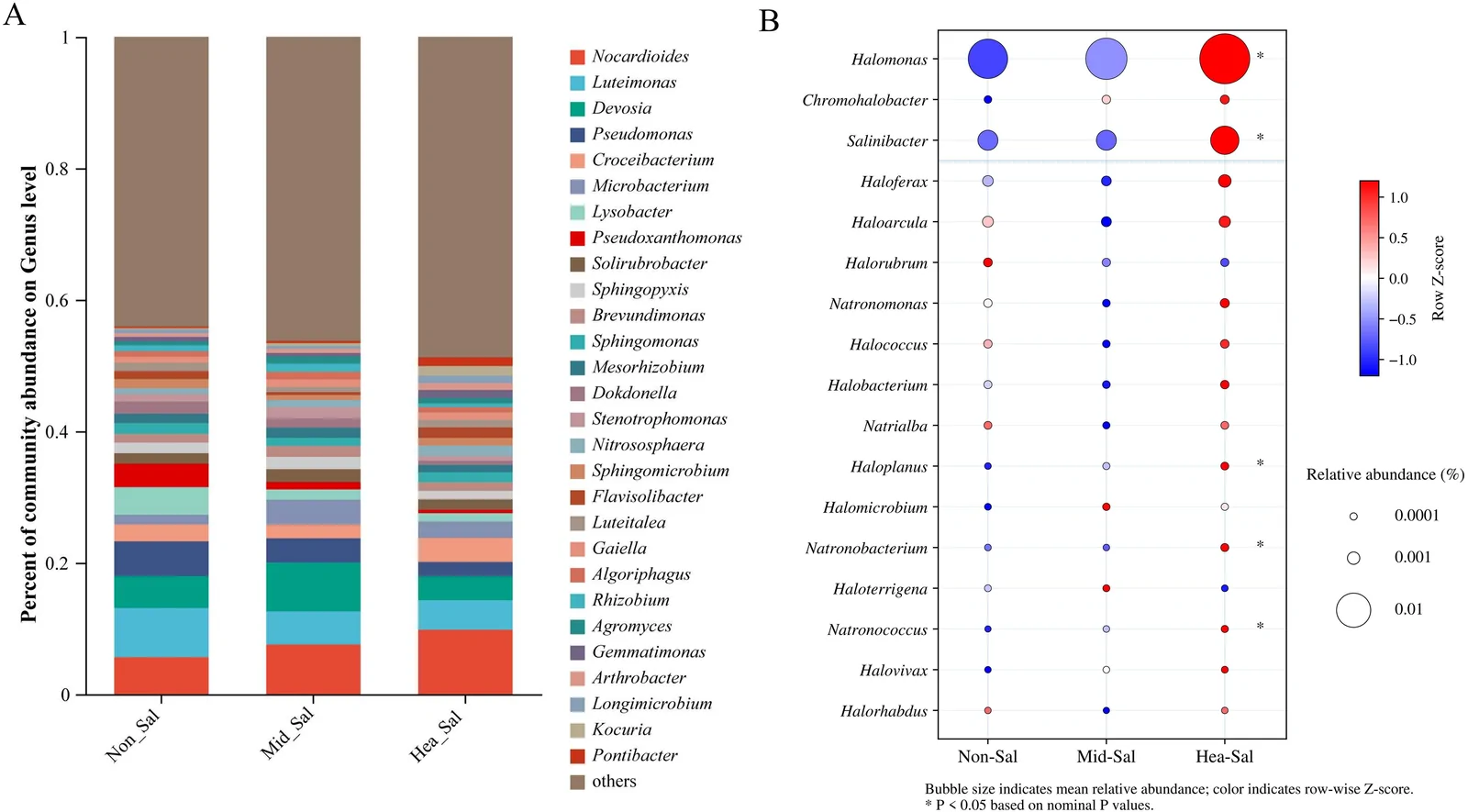
Genus-level composition and typical halophilic taxa of the wheat rhizosphere microbial community under different salinity treatments. Relative abundances of dominant rhizosphere microbial genera, defined as genera with a mean relative abundance >1% in at least one salinity treatment **(A)**. Bubble heatmap showing the relative abundance patterns of selected typical halophilic genera **(B)**. Bubble size represents mean relative abundance, whereas color represents row-wise Z-score-standardized abundance, with red and blue indicating relatively high and low abundances, respectively. Asterisks indicate genera showing differences among salinity treatments with FDR-adjusted P ≤ 0.10. Non-Sal, non-salinity treatment; Mid-Sal, moderate-salinity treatment; Hea-Sal, heavy-salinity treatment.

To further evaluate the response of well-established halophilic taxa to salinity, 17 representative genera were examined, including three halophilic bacterial genera (*Halomonas*, *Chromohalobacter*, and *Salinibacter*) and 14 haloarchaeal genera (**Figure 3B**; **Supplementary Table S3**). Among these taxa, five genera (*Halomonas*, *Chromohalobacter*, *Haloplanus*, *Natronococcus*, and *Halovivax*) showed a progressive increase in relative abundance from Non-Sal to Mid-Sal and Hea-Sal, whereas Halorubrum showed a decreasing trend and Halomicrobium and Haloterrigena peaked under Mid-Sal conditions. The remaining nine genera exhibited no clear monotonic response to salinity. In terms of abundance, *Halomonas* was the dominant halophilic genus and increased from 0.01344% in Non-Sal to 0.02192% in Hea-Sal. *Salinibacter*, the second most abundant taxon, increased from 0.00322% to 0.00679% under severe salinity. Among haloarchaeal genera, *Haloferax* and *Haloarcula* showed relatively higher abundances, while *Haloplanus*, *Natronococcus*, and *Natronobacterium* showed pronounced increases under Hea-Sal despite their low absolute abundances. However, none of the examined genera remained significant after multiple-testing correction at FDR < 0.05, although *Halomonas*, *Salinibacter, Natronococcus*, *Natronobacterium*, and *Haloplanus* showed marginal differences (raw P < 0.05; FDR = 0.083–0.099). These results indicate salinity-associated, taxon-specific shifts in the relative abundances of typical halophilic microorganisms, although the statistical evidence remained marginal after multiple-testing correction.

### 3.4 Salinity-associated redistribution of nutrient-related microbial functional potential

KEGG Orthology (KO)-based profiling indicated that the relative representation of genes associated with carbon, nitrogen, and sulfur metabolism differed across salinity treatments (**Figure 4**; **Supplementary Table S4**). In contrast, phosphorus-related functional potential changed only modestly, with differences mainly confined to specific KOs involved in phosphorus acquisition and turnover. Carbon metabolism exhibited clear pathway-level shifts (**Figure 4A**; **Supplementary Table S4**). In Hea-Sal, the relative abundances of KOs related to 6-phospho-beta-glucosidase (K01223) and fructose-1, 6-bisphosphatase III (K04041) increased by 8.00- and 3.93-fold, respectively, while the relative abundances of KOs related to cellulose synthase (K00694) increased by 1.81-fold, indicating enrichment of genes related to carbohydrate utilization, polysaccharide metabolism, and gluconeogenesis. By contrast, the relative abundances of KOs related to 2-methylcitrate synthase (K01659) and isocitrate dehydrogenase kinase/phosphatase (K00906) decreased by 51.0% and 45.0%, respectively, suggesting a redistribution among carbon metabolic routes under salinity stress.

**Figure 4:**
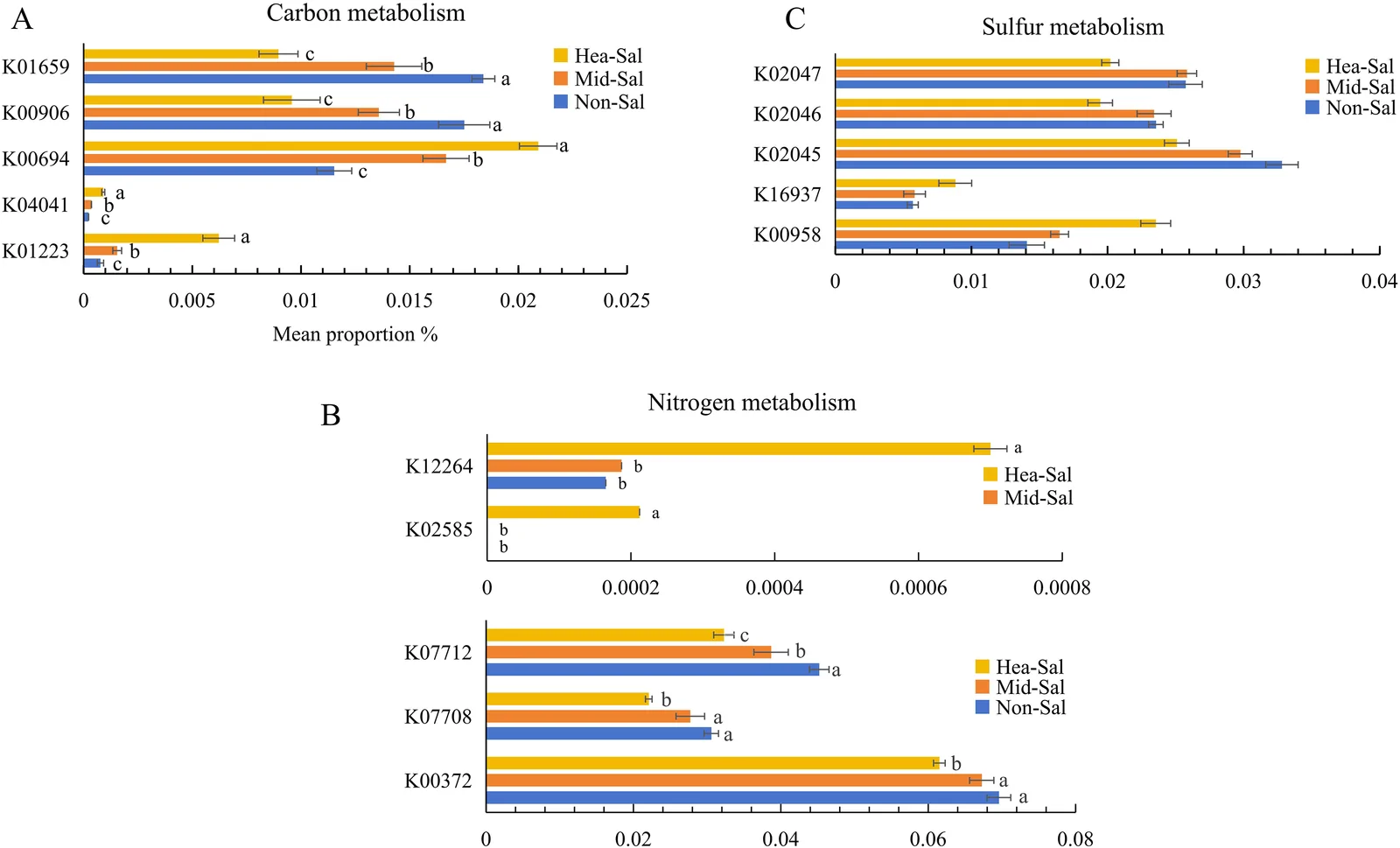
Salinity-associated changes in microbial functional potential related to carbon, nitrogen, and sulfur metabolism in the wheat rhizosphere. Relative abundances of selected KEGG orthologs (KOs) associated with **(A)** carbon metabolism, **(B)** nitrogen metabolism, and **(C)** sulfur metabolism under different salinity treatments. Values represent the mean relative abundances of the corresponding KOs. Non-Sal, non-salinity treatment; Mid-Sal, moderate-salinity treatment; Hea-Sal, heavy-salinity treatment.

The overall relative abundance of KOs related to nitrogen- (**Figure 4B**; **Supplementary Table S4**) and sulfur-metabolism (**Figure 4C**; **Supplementary Table S4**) decreased with increasing salinity, with reductions of 2.88% and 7.66%, respectively, in Hea-Sal relative to Non-Sal. Despite these overall declines, individual functional branches responded differently. Within nitrogen metabolism, the relative abundances of nitrogen fixation-related KOs nifK (K02591) and nifB (K02585) increased by 7.85- and 4.05-fold, whereas the relative abundances of KOs associated with assimilatory nitrate reductase (K00372) decreased by 11.6% and the NtrC-family regulatory components K07708 and K07712 decreased by 27.8% and 28.6%, respectively. Within sulfur metabolism, the relative abundances of KOs related to anaerobic sulfite reductase subunit B (K16951), sulfate adenylyltransferase (K00958), and thiosulfate dehydrogenase (K16937) increased by 8.68-, 1.67-, and 1.55-fold, respectively. In contrast, the relative abundances of KOs related to the NADPH-dependent sulfite reductase components K00380 and K00381 decreased by 44.9% and 22.3%, while the sulfate/thiosulfate ABC transport system KOs (K02045–K02047) decreased by 17.4–23.5%. Thus, although nitrogen and sulfur metabolism declined overall, their internal functional composition was markedly reorganized across nutrient acquisition, transformation, and assimilation processes.

### 3.5 Salinity was associated with greater representation of salt-adaptation-related functional potential

Compared with nutrient-related functions, the relative abundances of KOs related to salt adaptation showed a more coherent directional pattern across the salinity gradient (**Figure 5**; **Supplementary Table S5**). Relative abundances of KOs encoding compatible-solute synthesis and transport were particularly enriched. Accumulation of KOs involved in compatible solutes—the canonical microbial strategy to offset osmotic stress—was reinforced at both the biosynthetic and uptake levels. The relative abundances of KOs related to synthesis of ectoine (K06720, + 1.41-fold; **Figure 5**), glycine betaine (K17755 choline oxidase, +4.36-fold; K00130 betaine-aldehyde dehydrogenase, +1.28-fold), trehalose (K13057, + 1.72-fold; K05342, + 1.70-fold), mannitol (K00009, + 2.07-fold) and inositol (K01858, + 1.43-fold) all increased. The largest fold change was observed for the trehalose permease KO K17313, whose relative abundance increased 15.67-fold, and transporter KOs for glycine betaine and proline (K02168, + 1.75-fold; K03451, + 1.60-fold; K03762, + 1.38-fold) as well as the osmoprotectant ABC system KOs (K05845–K05847, +1.5–1.6-fold) were likewise enriched.

**Figure 5:**
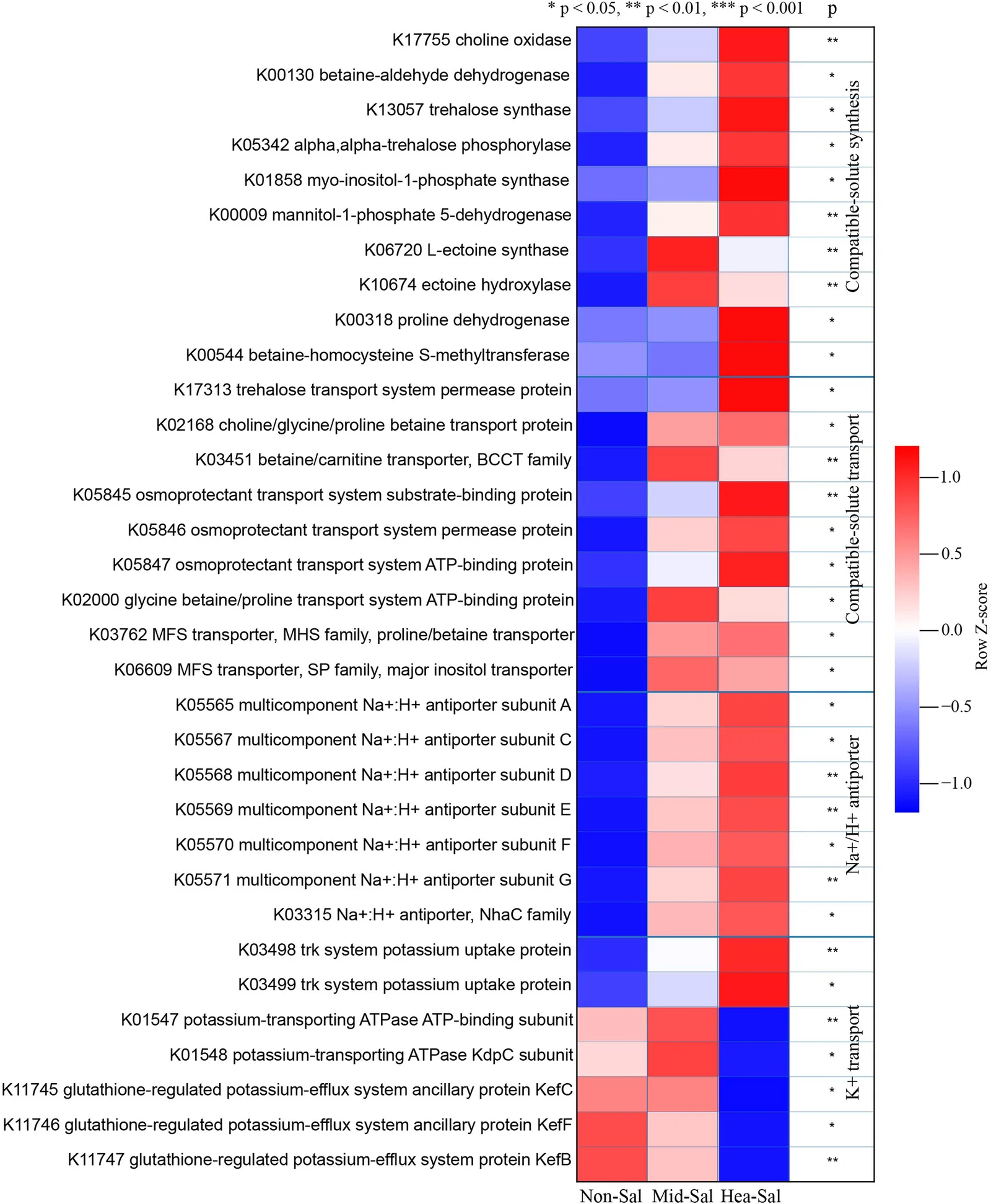
Heatmap of salinity-associated KEGG orthologs related to osmotic adaptation and ion homeostasis. The heatmap shows the relative abundance patterns of selected KEGG orthologs (KOs) across the Non-Sal, Mid-Sal, and Hea-Sal treatments. KO abundances were standardized by row-wise Z-score transformation based on the mean abundance of each KO across the three salinity treatments, with red indicating relatively high abundance and blue indicating relatively low abundance. The selected KOs were grouped into four functional categories: compatible-solute synthesis, compatible-solute transport, Na^+^/H^+^ antiporter, and K^+^ transport. Asterisks indicate significant differences among salinity treatments (p < 0.05, p < 0.01, and p < 0.001).

KOs associated with ion homeostasis showed a coordinated pattern consistent with increased encoded potential for Na^+^ export and K^+^ retention. The relative abundances of KOs related to the Mrp-type Na^+^/H^+^ antiporter (K05565–K05571) increased by 1.53–1.92-fold, together with NhaC (K03315, + 1.72-fold), and KOs encoding the V/A-type H^+^/Na^+^-translocating ATPase were upregulated across eight subunits (K02117–K02123, K02107; +1.4–2.5-fold), consistent with greater encoded potential for Na^+^ extrusion. Simultaneously, the relative abundances of K^+^-retention-related KOs showed a reciprocal pattern: KOs of the Trk K^+^-uptake system (K03498, K03499; +1.47–1.51-fold) increased, whereas KOs of the Kdp high-affinity import system (K01547, K01548; −32% to −34%) and the glutathione-regulated K^+^-efflux system Kef (K11745–K11747; −37% to −66%) declined, preventing futile K^+^ loss. This reciprocal upregulation of Na^+^-exporter KOs and downregulation of K^+^-efflux KOs mirrors the strategy of halophilic and halotolerant bacteria.

### 3.6 Field remediation reversed soil deterioration and reduced WCR severity

The independent field remediation experiment showed that replacing saline groundwater with non-saline irrigation substantially improved soil physicochemical conditions. Compared with Con-Sal, SSC decreased from 3.28 ± 0.05 to 1.39 ± 0.03 g kg^-^¹ in Rem-Sal, representing a 57.6% reduction, while soil pH decreased from 8.05 ± 0.04 to 7.79 ± 0.04. Nutrient availability also improved following remediation: AP increased by 28.1% (27.53 ± 0.28 to 35.26 ± 0.32 mg kg^-^¹), TN by 3.35% (2.69 ± 0.05 to 2.78 ± 0.04 g kg^-^¹), and AK by 23.3% (798.25 ± 15.64 to 984.25 ± 15.36 mg kg^-^¹). SOC increased slightly from 41.98 ± 0.11 to 42.58 ± 0.13 g kg^-^¹, but the difference was not significant.

Improvement in soil conditions was accompanied by a marked reduction in WCR severity. The disease index decreased from 53.24 in Con-Sal to 27.06 in Rem-Sal, corresponding to a 49.17% reduction. These results provide independent field evidence that alleviating soil salinity can restore key physicochemical properties and substantially reduce the associated disease severity.

## 4 Discussion

### 4.1 Long-term salinity stress is associated with soil physicochemical degradation

Long-term saline-water irrigation was associated with a marked decline in soil fertility. Under severe salinity, TN, AP, and AK decreased substantially, with AP showing the largest reduction. Salt accumulation can alter soil ionic balance and nutrient retention, while the accompanying increase in pH further constrains nutrient availability (Hassani et al., 2020). This effect is particularly important for phosphorus because alkalization can alter P sorption and precipitation equilibria, reducing the fraction available to plants (Li et al., 2025). Changes in microbial nutrient cycling may also contribute to this decline. Nitrogen- and sulfur-metabolism-related functions decreased overall under severe salinity, together with reductions in several functions involved in nutrient uptake and assimilation. Similar shifts in microbial nutrient-cycling potential have been reported in saline soils (Li et al., 2024). Because phosphorus-related microbial functions changed only modestly in our dataset, however, the pronounced loss of AP was more likely driven primarily by soil geochemical constraints than by microbial functional changes alone. Nevertheless, microbially mediated phosphorus cycling could also contribute to P availability, and the relative contributions of geochemical versus microbial processes cannot be fully separated with the present data.

The deterioration of soil conditions was accompanied by a strong increase in wheat crown rot (WCR), with the disease index rising from 10.51 under non-saline conditions to 32.88 under severe salinity. Salinity imposes both osmotic and ionic stress on plants, and the concurrent depletion of N, P, and K may further reduce the capacity of wheat to tolerate pathogen infection. Nitrogen availability has been shown to influence the severity of Fusarium crown rot and its interaction with wheat physiology (Buster et al., 2023). Drought, another stress that limits plant water status, can also intensify crown rot and shares part of the wheat stress-response network activated during Fusarium infection (Su et al., 2024). However, the present study did not quantify pathogens or infection assays. We therefore cannot distinguish whether greater WCR severity resulted from increased pathogen pressure, increased host susceptibility, reduced microbiome-mediated suppression, or a combination of these processes. The increase in WCR observed here is therefore more plausibly viewed as a consequence of the broader deterioration of the rhizosphere environment rather than the effect of any single soil property.

### 4.2 Long-term salinity was associated with rhizosphere microbiome restructuring consistent with environmental filtering

Microbial community composition was more responsive to salinity than alpha diversity. Moderate salinity was associated with little change in the Chao and Shannon indices, and even under severe salinity the reductions in richness and diversity were relatively modest. In contrast, salinity substantially altered genus-level community composition. The number of dominant genera increased from 18 in Non-Sal to 20 in Mid-Sal and 22 in Hea-Sal, while their relative contributions changed markedly across the salinity gradient. These results indicate that chronic salinity primarily reorganized the relative abundance structure of the rhizosphere microbial community rather than causing a general loss of dominant taxa. The responses of salt-adapted genera further support the role of environmental filtering in rhizosphere community assembly. Among the 17 representative halophilic genera examined, five genera (*Halomonas*, *Chromohalobacter*, *Haloplanus*, *Natronococcus*, and *Halovivax*) increased progressively with salinity, whereas *Halorubrum* decreased, *Halomicrobium* and Haloterrigena peaked under moderate salinity, and the remaining nine genera showed no clear monotonic response. These taxon-specific patterns indicate that salt adaptation does not necessarily lead to uniform enrichment, but rather reflects differences in salinity tolerance and ecological niche preference. Rhizosphere community assembly is jointly governed by host-mediated selection and soil environmental constraints (Trivedi et al., 2020). Under persistent salinity, the abiotic filter is likely to become stronger, favoring taxa with physiological traits that permit growth under high osmotic pressure and ionic stress. Plant-mediated recruitment can still influence rhizosphere assembly, but its outcome is inevitably constrained by the physicochemical environment in which roots and microorganisms interact (Yang et al., 2025). Thus, the major ecological response observed here was a selective replacement of community members, with the modest decline in diversity appearing to be a secondary consequence.

### 4.3 Salinity was associated with microbial functional reorganization and increased representation of salt-adaptation functions

Changes in community composition were accompanied by substantial redistribution of metagenomic functional potential. KOs related to carbon metabolism showed mixed responses, and the nitrogen- and sulfur-metabolism-related KOs declined overall by 2.88% and 7.66%, respectively, while several specific branches increased. For example, nifK and nifB were enriched, whereas assimilatory nitrate reduction and NtrC-related regulatory functions declined. KOs related to sulfur metabolism showed a similar divergence, with enrichment of selected reductive and thiosulfate-related functions but decreases in sulfate transport and assimilatory pathways. Such contrasting responses indicate that salinity did not simply suppress the potential for nutrient metabolism. Instead, it altered the potential for different nutrient acquisition and transformation routes. This is consistent with observations that microbial genes involved in C, N, and S cycling differ substantially in their sensitivity to salinity (Li et al., 2024).

Genes involved in compatible-solute synthesis and transport were broadly over-represented, together with components of Mrp-type Na^+^/H^+^ antiporters and V/A-type H^+^/Na^+^-ATPases. These systems address different consequences of salt exposure: compatible solutes help maintain cellular osmotic balance, ion transport systems limit intracellular Na^+^ accumulation and preserve electrochemical gradients, whereas stress-response pathways protect membranes and macromolecules from secondary damage. Their simultaneous enrichment suggests that long-term salinity imposed a strong and persistent selective pressure on microbial survival strategies. The resulting functional profile may reflect both physiological plasticity within resident microorganisms and the increasing dominance of salt-tolerant taxa carrying distinct functional repertoires.

This distinction is also important for plant health. A microbiome that is better adapted to a saline environment is not necessarily more beneficial to its host. Under severe salinity, microbial functions associated with osmotic and ionic stress tolerance were strongly enriched, yet several nutrient-cycling functions declined and WCR severity increased. Rhizosphere microorganisms can suppress soil-borne pathogens through nutrient competition and the production of antimicrobial compounds (Berendsen et al., 2012). They can also contribute more broadly to plant health and disease resistance through community-level interactions with the host (Banerjee and van der Heijden, 2023). The increase in WCR is therefore most appropriately interpreted as occurring alongside simultaneous changes in the plant environment, soil nutrient status, and rhizosphere microbiome. Direct tests of microbiome-mediated disease effects would require pathogen quantification, microbiome manipulation, longitudinal sampling, or mediation/path analyses.

### 4.4 Irrigation conversion alleviated soil degradation and reduced WCR severity

The 3-year irrigation-conversion experiment provided complementary evidence from the opposite direction. Replacing saline irrigation with non-saline water reduced soil salinity and pH, improved nutrient availability, and decreased WCR severity by 49.17%. The response was broadly opposite to that observed along the long-term salinity gradient, supporting the view that sustained salt accumulation contributes to both soil deterioration and increased disease risk, while at least part of these effects can be alleviated by reducing external salt inputs. Similar benefits of improved irrigation and salinity management have been reported in wheat-based systems (Su et al., 2021; Liu et al., 2024). Although the remediation experiment did not include microbiome profiling, improved soil conditions may provide a more favorable basis for the recovery of plant-beneficial microbial functions. Whether microbial community structure and functional potential recover in parallel with soil physicochemical properties remains to be tested through longitudinal microbiome and activity-based analyses.

### 4.5 Novelty, limitations, and future directions

Our study lies in the combination of a 19-year controlled salinity gradient with an independent 3-year remediation demonstration, which provides long-term field evidence that chronic salinization is associated with coordinated soil–microbiome–disease changes and that the soil and disease responses are at least partly reversible under practical management. Nevertheless, these findings should be interpreted with some caution, as several key questions remain open. In future work, microbiome profiling will be performed in the remediation experiment, to better reveal whether the rhizosphere microbial community and its functional potential recover together with the soil and disease responses. And quantitative detection of pathgeons (e.g., by qPCR of *F. pseudograminearum*) will be performed to better reveal how pathogen abundance responds to salinity and irrigation conversion and how this relates to disease severity. Combined with manipulative experiments such as microbiome transplantation along the same salinity gradient, these approaches are expected to allow the proposed salinity–soil–microbe–disease mechanism to be examined in greater depth and to clarify how long-term salinity shapes soil health and disease dynamics in agricultural ecosystems.

## 5 Conclusions

Long-term salinity was associated with coordinated deterioration of soil fertility, restructuring of rhizosphere microbial community composition, redistribution of metagenomic functional potential, and increased WCR severity. Rather than demonstrating a complete microbiome-mediated causal pathway, the 19-year gradient experiment provides long-term field evidence that these responses occur together across increasing salinity. The independent 3-year irrigation-conversion experiment further showed that reducing external salt inputs improved soil physicochemical conditions and substantially decreased disease severity, demonstrating management responsiveness of the soil and disease components. Because the remediation experiment did not include microbiome profiling, microbiome recovery remains unverified. Overall, the combined experiments support restoration of favorable soil conditions as a practical component of WCR risk reduction in salt-affected wheat systems while identifying microbiome recovery, pathogen dynamics, and causal mediation as priorities for future work.

## Data Availability

The data presented in this study are openly available in the NCBI Sequence Read Archive under the accession number PRJNA1414827.
