# Supplementary material for: Long-term salinity is associated with deteriorated soil properties, rhizosphere microbiome restructuring, and elevated wheat crown rot: evidence from a 19-year gradient experiment and a 3-year remediation verification
Source: Front Plant Sci. 2026 Oct 23;17:1992882. doi: 10.3389/fpls.2026.1992882 (PMC13739487; doi:10.3389/fpls.2026.1992882)
Supplement: Supplementary file 1 [file DataSheet1.doc]

**Long-term salinity is associated with deteriorated soil properties, rhizosphere microbiome restructuring, and elevated wheat crown rot: Evidence from a 19-year gradient experiment and a 3-year remediation verification**

Yajiao Wang^1^, Sen Han^1^, Xuetong Liu^2^, Hongkai Dang^2^, Chunlian Zheng^2^, Yudong Zheng^2^, Caiyun Cao^2*^, Yuxing Wu^1*^

^1^ Plant Protection Institute, Hebei Academy of Agriculture and Forestry Sciences/National Collection of Plant-associated Microbes (Hebei)/IPM Innovation Center of Hebei Province/ International Science and Technology Joint Research Center on IPM of Hebei Province, Baoding 071000, China

^2^ Dryland Farming Institute of Hebei Academy of Agricultural and Forestry Sciences, Hengshui 053000, China

*Corresponding authors: Yuxing Wu ([wyx1209@163.com](mailto:konglingxiao163@163.com)); Caiyun Cao (cycao1234@126.com)

Table S1. Raw plant counts, disease-grade distributions, and wheat crown rot disease indices for each biological replicate

| **Treatment** | **Replicate** | **Total Plants** | **Disease Grade 0** | **Disease Grade 1** | **Disease Grade 2** | **Disease Grade 3** | **Disease Grade 4** | **Disease Index** | **Average Disease Index** | **Deviation** |
| --- | --- | --- | --- | --- | --- | --- | --- | --- | --- | --- |
| Non-Sal | 1 | 99 | 66 | 29 | 4 | 0 | 0 | 9.34 | 10.51 | 1.05 |
|  | 2 | 99 | 61 | 31 | 7 | 0 | 0 | 11.36 |  |  |
|  | 3 | 97 | 62 | 29 | 5 | 1 | 0 | 10.82 |  |  |
| Mid-Sal | 1 | 100 | 28 | 41 | 22 | 6 | 3 | 28.75 | 28.10 | 1.58 |
|  | 2 | 100 | 25 | 42 | 25 | 7 | 1 | 29.25 |  |  |
|  | 3 | 96 | 28 | 40 | 23 | 5 | 0 | 26.30 |  |  |
| Hea-Sal | 1 | 100 | 21 | 39 | 26 | 10 | 4 | 34.25 | 32.88 | 2.36 |
|  | 2 | 100 | 18 | 39 | 32 | 10 | 1 | 34.25 |  |  |
|  | 3 | 97 | 22 | 39 | 30 | 6 | 0 | 30.15 |  |  |
| Con-Sal | 1 | 100 | 5 | 25 | 38 | 25 | 7 | 51.00 | 53.24 | 3.25 |
|  | 2 | 100 | 6 | 22 | 38 | 27 | 7 | 51.75 |  |  |
|  | 3 | 97 | 3 | 18 | 35 | 31 | 10 | 56.96 |  |  |
| Rem-Sal | 1 | 100 | 30 | 42 | 24 | 4 | 0 | 25.50 | 27.06 | 1.89 |
|  | 2 | 99 | 29 | 42 | 22 | 5 | 1 | 26.52 |  |  |
|  | 3 | 96 | 24 | 41 | 23 | 7 | 1 | 29.17 |  |  |

Table S2. Relative abundances of major rhizosphere microbial genera across the long-term salinity gradient

| **Genus** | **Non-Sal_mean(%)** | **Non-Sal_sd(%)** | **Mid-Sal_mean(%)** | **Mid-Sal_sd(%)** | **Hea-Sal_mean(%)** | **Hea-Sal_sd(%)** | **p value** | **q value** |
| --- | --- | --- | --- | --- | --- | --- | --- | --- |
| *Devosia* | 4.84800 | 0.56300 | 7.45500 | 0.34200 | 3.66600 | 0.50200 | 0.01729 | 0.08331 |
| *Pseudoxanthomonas* | 3.56900 | 0.75100 | 1.11800 | 0.01600 | 0.50700 | 0.09300 | 0.00728 | 0.08331 |
| *Microbacterium* | 1.49000 | 0.11900 | 3.79700 | 0.13600 | 2.51700 | 0.42200 | 0.01832 | 0.08331 |
| *Croceibacterium* | 2.54800 | 0.14400 | 2.07300 | 0.32100 | 3.62400 | 0.22900 | 0.0154 | 0.08331 |
| *Dokdonella* | 1.85100 | 0.23000 | 1.41200 | 0.18500 | 0.63800 | 0.05700 | 0.00728 | 0.08331 |
| *Sphingomonas* | 1.63400 | 0.07500 | 1.21000 | 0.21700 | 1.51900 | 0.02500 | 0.00728 | 0.08331 |
| *Pontibacter* | 0.23600 | 0.01200 | 0.33300 | 0.07000 | 1.33400 | 0.25800 | 0.00728 | 0.08331 |
| *Rhizobium* | 0.90600 | 0.14800 | 1.24000 | 0.06100 | 0.54500 | 0.04400 | 0.00728 | 0.08331 |
| *Steroidobacter* | 0.86900 | 0.22200 | 0.54200 | 0.06800 | 0.20200 | 0.04300 | 0.00728 | 0.08331 |
| *Longimicrobium* | 0.58900 | 0.08800 | 0.43400 | 0.02800 | 1.11000 | 0.25700 | 0.04352 | 0.1113 |
| *Gemmatimonas* | 0.70000 | 0.13200 | 0.48400 | 0.03100 | 1.19800 | 0.26400 | 0.0366 | 0.1016 |
| *Kocuria* | 0.16800 | 0.01400 | 0.39900 | 0.10200 | 1.49200 | 0.15200 | 0.00728 | 0.08331 |
| *Stenotrophomonas* | 1.14500 | 0.23800 | 1.74200 | 0.11400 | 0.69000 | 0.30900 | 0.00971 | 0.08331 |
| *Sphingomicrobium* | 1.39800 | 0.17400 | 0.75400 | 0.09800 | 1.14800 | 0.14600 | 0.0154 | 0.08331 |
| *Mesorhizobium* | 1.41400 | 0.06900 | 1.55200 | 0.08900 | 1.07700 | 0.25300 | 0.2364 | 0.3504 |
| *Lysobacter* | 4.20300 | 0.28300 | 1.54800 | 0.15300 | 1.27600 | 0.11900 | 0.00971 | 0.08331 |
| *Arthrobacter* | 0.55200 | 0.05500 | 0.65800 | 0.09100 | 1.04600 | 0.19600 | 0.02095 | 0.08331 |
| *Kosakonia* | 0.02700 | 0.01200 | 0.04100 | 0.00700 | 0.77100 | 0.66700 | 0.01247 | 0.08331 |
| *Sphingopyxis* | 1.61500 | 0.19900 | 1.88600 | 0.36400 | 1.26900 | 0.01300 | 0.0154 | 0.08331 |
| *Flavisolibacter* | 1.22700 | 0.11600 | 0.42900 | 0.05700 | 1.60600 | 0.43300 | 0.02307 | 0.08331 |
| *Luteimonas* | 7.48300 | 1.09400 | 5.02800 | 0.50200 | 4.49100 | 0.79300 | 0.01729 | 0.08331 |
| *Nitrososphaera* | 0.87600 | 0.15200 | 1.07000 | 0.13200 | 1.63000 | 0.40500 | 0.03078 | 0.09132 |
| *Luteitalea* | 1.26100 | 0.33000 | 0.79100 | 0.06200 | 1.14600 | 0.07000 | 0.02491 | 0.08331 |
| *Nocardioides* | 5.64500 | 0.75600 | 7.57300 | 0.09500 | 9.80200 | 2.74600 | 0.04884 | 0.1204 |
| *Variovorax* | 0.99400 | 0.14100 | 0.89000 | 0.01100 | 0.43300 | 0.08600 | 0.02444 | 0.08331 |
| *Algoriphagus* | 0.82300 | 0.16700 | 1.18400 | 0.15100 | 0.81500 | 0.16700 | 0.02491 | 0.08331 |
| *Aeromicrobium* | 0.64600 | 0.04700 | 0.88900 | 0.09500 | 0.89500 | 0.16000 | 0.0366 | 0.1016 |
| *Agromyces* | 0.58800 | 0.16700 | 1.11100 | 0.03800 | 0.85500 | 0.28700 | 0.116 | 0.2091 |
| *Pseudomonas* | 5.29300 | 2.38700 | 3.67800 | 0.32500 | 2.17500 | 1.53400 | 0.08368 | 0.1696 |
| *Brevundimonas* | 1.35400 | 0.22700 | 1.65900 | 0.30300 | 1.34800 | 0.35100 | 0.09212 | 0.1792 |
| *Gaiella* | 0.91900 | 0.29600 | 1.13700 | 0.04800 | 1.12900 | 0.28900 | 0.2977 | 0.4082 |
| *Solirubrobacter* | 1.58100 | 0.52900 | 1.96700 | 0.13900 | 1.59900 | 0.55100 | 0.8411 | 0.8694 |

Note: Values are presented as the mean relative abundance ± standard deviation (SD) of three biological replicates. Non-Sal, non-salinity treatment; Mid-Sal, moderate-salinity treatment; Hea-Sal, heavy-salinity treatment. P values indicate the overall differences in relative abundance among the three salinity treatments. Statistical significance was defined as p < 0.05, q < 0.05, whereas 0.05 ≤ q < 0.10 was interpreted as marginal evidence.

Table S3. Relative abundances of selected typical halophilic genera in the wheat rhizosphere under different salinity treatments.

| **Genus** | **Non-Sal_mean(%)** | **Non-Sal_sd(%)** | **Mid-Sal_mean(%)** | **Mid-Sal_sd(%)** | **Hea-Sal_mean(%)** | **Hea-Sal_sd(%)** | **p value** | **q value** |
| --- | --- | --- | --- | --- | --- | --- | --- | --- |
| *Halomonas* | 0.01344 | 0.00109 | 0.01479 | 0.00105 | 0.02192 | 0.00276 | 0.0154 | 0.08331 |
| *Chromohalobacter* | 0.00016 | 0.00015 | 0.00029 | 0.00008 | 0.00036 | 0.00013 | 0.1462 | 0.2454 |
| *Salinibacter* | 0.00322 | 0.00071 | 0.0032 | 0.00054 | 0.00679 | 0.00132 | 0.02491 | 0.08331 |
| *Haloferax* | 0.00066 | 0.00019 | 0.0005 | 0.00017 | 0.00106 | 0.00037 | 0.09212 | 0.1792 |
| *Haloarcula* | 0.0007 | 0.00022 | 0.00054 | 0.00013 | 0.00078 | 0.00028 | 0.3679 | 0.4612 |
| *Halobacterium* | 0.00022 | 0.00012 | 0.00018 | 0.00009 | 0.00029 | 0.00007 | 0.4374 | 0.5267 |
| *Halococcus* | 0.00027 | 0.0001 | 0.00016 | 0.00006 | 0.00031 | 0.00011 | 0.1054 | 0.1993 |
| *Halorubrum* | 0.00034 | 0.00004 | 0.00026 | 0.00005 | 0.00025 | 0.00009 | 0.1229 | 0.2143 |
| *Haloterrigena* | 0.00006 | 0.00005 | 0.00008 | 0.00006 | 0.00005 | 0.00001 | 0.2977 | 0.4082 |
| *Halomicrobium* | 0.00006 | 0.00006 | 0.00013 | 0.00006 | 0.0001 | 0.00007 | 0.2457 | 0.3535 |
| *Haloplanus* | 0.00004 | 0.00002 | 0.00009 | 0.00002 | 0.0002 | 0.00016 | 0.03455 | 0.0988 |
| *Halorhabdus* | 0.00002 | 0.00001 | 0.00001 | 0.00001 | 0.00002 | 0.00003 | 0.2833 | 0.3981 |
| *Halovivax* | 0.00002 | 0.00004 | 0.00004 | 0.00007 | 0.00006 | 0.00005 | 0.5688 | 0.6468 |
| *Natrialba* | 0.00019 | 0.0001 | 0.00011 | 0.00005 | 0.00019 | 0.00003 | 0.2977 | 0.4082 |
| *Natronomonas* | 0.00027 | 0.00014 | 0.00017 | 0.00006 | 0.00038 | 0.00017 | 0.1183 | 0.2106 |
| *Natronococcus* | 0 | 0 | 0.00003 | 0.00004 | 0.0001 | 0.00009 | 0.02308 | 0.08331 |
| *Natronobacterium* | 0.00004 | 0.00003 | 0.00003 | 0.00003 | 0.00022 | 0.00011 | 0.02319 | 0.08331 |

**Note:** Values are presented as the mean relative abundance ± standard deviation (SD) of three biological replicates. Non-Sal, non-salinity treatment; Mid-Sal, moderate-salinity treatment; Hea-Sal, heavy-salinity treatment. P values indicate the overall differences among salinity treatments. Statistical significance was defined as p < 0.05, q < 0.05, whereas 0.05 ≤ q < 0.10 was interpreted as marginal evidence.

Table S4. Relative abundances of selected KEGG orthologs associated with carbon, nitrogen, and sulfur metabolism under different salinity treatments.

|  | KO | Function | Non-Sal mean | Non-Sal sd | Mid-Sal mean | Mid-Sal_sd | Hea-Sal mean | Hea-Sal_sd | p value | p value |
| --- | --- | --- | --- | --- | --- | --- | --- | --- | --- | --- |
| Carbon metabolism | K01223 | 6-phospho-beta-glucosidase | 0.000777 | 0.000142 | 0.001546 | 0.000202 | 0.006217 | 0.000732 | 0.00728 | 0.01981 |
|  | K04041 | fructose-1,6-bisphosphatase III | 0.00023 | 6.4e-05 | 0.000357 | 7e-05 | 0.000903 | 0.000071 | 0.00971 | 0.01981 |
|  | K00694 | cellulose synthase (UDP-forming) | 0.01153 | 0.000801 | 0.01667 | 0.001062 | 0.02091 | 0.000863 | 0.00728 | 0.01981 |
|  | K00906 | isocitrate dehydrogenase kinase/phosphatase | 0.01751 | 0.001176 | 0.01358 | 0.000946 | 0.009579 | 0.001302 | 0.00971 | 0.01981 |
|  | K01659 | 2-methylcitrate synthase | 0.01839 | 0.000524 | 0.01428 | 0.001276 | 0.008963 | 0.000896 | 0.01247 | 0.01981 |
| nitrogen metabolism | K02585 | nitrogen fixation protein NifB | 5.2e-05 | 3.3e-05 | 9.6e-05 | 5.2e-05 | 0.000212 | 8e-05 | 0.02178 | 0.02662 |
|  | K12264 | anaerobic nitric oxide reductase flavorubredoxin | 0.000165 | 6.8e-05 | 0.000187 | 5e-05 | 0.0007 | 0.000023 | 0.02307 | 0.02662 |
|  | K00372 | assimilatory nitrate reductase catalytic subunit | 0.06959 | 0.001609 | 0.06726 | 0.001646 | 0.06151 | 0.000788 | 0.01247 | 0.01981 |
|  | K07708 | two-component system, NtrC family, nitrogen regulation sensor histidine kinase | 0.03057 | 0.000977 | 0.02772 | 0.001941 | 0.02208 | 0.000448 | 0.00971 | 0.01981 |
|  | K07712 | two-component system, NtrC family, nitrogen regulation response regulator GlnG | 0.04522 | 0.001322 | 0.03868 | 0.002332 | 0.03227 | 0.001376 | 0.01321 | 0.01981 |
| sulfur metabolism | K00958 | sulfate adenylyltransferase | 0.01407 | 0.001295 | 0.01648 | 0.000657 | 0.02354 | 0.001096 | 0.00971 | 0.01981 |
|  | K16937 | thiosulfate dehydrogenase (quinone) large subunit | 0.005688 | 0.000403 | 0.005822 | 0.000796 | 0.008815 | 0.001204 | 0.02307 | 0.02662 |
|  | K02045 | sulfate/thiosulfate transport system ATP-binding protein | 0.03281 | 0.001189 | 0.02975 | 0.000882 | 0.02509 | 0.000911 | 0.00728 | 0.01981 |
|  | K02046 | sulfate/thiosulfate transport system permease protein | 0.02355 | 0.000536 | 0.02342 | 0.001249 | 0.01946 | 0.000904 | 0.03731 | 0.03731 |
|  | K02047 | sulfate/thiosulfate transport system permease protein | 0.02573 | 0.00123 | 0.02583 | 0.000714 | 0.0202 | 0.00063 | 0.02491 | 0.02669 |

Note: Values represent the mean relative abundance ± standard deviation (SD) of KEGG orthologs (KOs) across three biological replicates. KOs were assigned according to the KEGG database and grouped into carbon, nitrogen, and sulfur metabolism according to their annotated functions. Non-Sal, non-salinity treatment; Mid-Sal, moderate-salinity treatment; Hea-Sal, heavy-salinity treatment. p< 0.05 , q < 0.05 was considered statistically significant.

Table S5. Relative abundances of KEGG orthologs involved in microbial osmotic adjustment and ion homeostasis under different salinity treatments.

| Functional category | KO | Function | Non-Sal mean | Non-Sal sd | Mid-Sal mean | Mid-Sal_sd | Hea-Sal mean | Hea-Sal_sd | p value | q value |
| --- | --- | --- | --- | --- | --- | --- | --- | --- | --- | --- |
| Compatible-solute synthesis | K17755 | choline oxidase | 0.000765 | 0.000148 | 0.001645 | 0.000266 | 0.003336 | 0.000577 | 0.00728 | 0.02670 |
|  | K00130 | betaine-aldehyde dehydrogenase | 0.04741 | 0.002009 | 0.05516 | 0.006238 | 0.06068 | 0.006461 | 0.02962 | 0.03055 |
|  | K13057 | trehalose synthase | 0.008021 | 0.001136 | 0.00981 | 0.002275 | 0.01383 | 0.002694 | 0.04884 | 0.04884 |
|  | K05342 | alpha,alpha-trehalose phosphorylase | 0.01632 | 0.00279 | 0.02289 | 0.004711 | 0.02771 | 0.003764 | 0.02962 | 0.03055 |
|  | K01858 | myo-inositol-1-phosphate synthase | 0.03097 | 0.003597 | 0.03251 | 0.004696 | 0.0444 | 0.00556 | 0.02307 | 0.02820 |
|  | K00009 | mannitol-1-phosphate 5-dehydrogenase | 0.002685 | 0.000488 | 0.004271 | 0.000818 | 0.005545 | 0.00032 | 0.00971 | 0.02670 |
|  | K06720 | L-ectoine synthase | 0.001283 | 0.000105 | 0.002479 | 0.000248 | 0.001815 | 0.000263 | 0.00728 | 0.02670 |
|  | K10674 | ectoine hydroxylase | 0.004685 | 0.000552 | 0.007519 | 0.000393 | 0.006456 | 0.000395 | 0.00728 | 0.02670 |
|  | K00318 | proline dehydrogenase | 0.01811 | 0.00134 | 0.01855 | 0.00144 | 0.02548 | 0.000338 | 0.02307 | 0.02820 |
|  | K00544 | betaine-homocysteine S-methyltransferas | 0.002131 | 0.000488 | 0.001993 | 0.000324 | 0.003892 | 0.000764 | 0.02444 | 0.02835 |
| Compatible-solute transport | K17313 | trehalose transport system permease protein | 9e-06 | 1.8e-05 | 1.8e-05 | 3.6e-05 | 0.000138 | 2.6e-05 | 0.01481 | 0.02748 |
|  | K02168 | choline/glycine/proline betaine transport protein | 0.019 | 0.002459 | 0.03149 | 0.001338 | 0.03328 | 0.001809 | 0.01832 | 0.02748 |
|  | K03451 | betaine/carnitine transporter, BCCT family | 0.004296 | 0.000663 | 0.008256 | 0.00044 | 0.006883 | 0.000708 | 0.00728 | 0.02670 |
|  | K05845 | osmoprotectant transport system substrate-binding protein | 0.02464 | 0.000921 | 0.02967 | 0.003454 | 0.03908 | 0.002324 | 0.00971 | 0.02670 |
|  | K05846 | osmoprotectant transport system permease protein | 0.0232 | 0.000485 | 0.03278 | 0.004441 | 0.03742 | 0.004493 | 0.01832 | 0.02748 |
|  | K05847 | osmoprotectant transport system ATP-binding protein | 0.02236 | 0.001768 | 0.0272 | 0.003497 | 0.0333 | 0.003008 | 0.01321 | 0.02725 |
|  | K02000 | glycine betaine/proline transport system ATP-binding protein | 0.0223 | 0.002876 | 0.03197 | 0.002058 | 0.02839 | 0.002969 | 0.01247 | 0.02725 |
|  | K03762 | MFS transporter, MHS family, proline/betaine transporter | 0.01269 | 0.000798 | 0.01701 | 0.000753 | 0.01748 | 0.001097 | 0.02307 | 0.02820 |
|  | K06609 | MFS transporter, SP family, major inositol transporter | 0.000499 | 0.000102 | 0.001026 | 0.000246 | 0.000944 | 0.000163 | 0.02307 | 0.02820 |
| Na+/H+ antiporter | K05565 | multicomponent Na+:H+ antiporter subunit A | 0.03051 | 0.000834 | 0.04264 | 0.005107 | 0.04883 | 0.004367 | 0.01247 | 0.02725 |
|  | K05567 | multicomponent Na+:H+ antiporter subunit C | 0.005817 | 0.000522 | 0.008049 | 0.000829 | 0.008894 | 0.001461 | 0.02307 | 0.02820 |
|  | K05568 | multicomponent Na+:H+ antiporter subunit D | 0.02532 | 0.001701 | 0.03403 | 0.003882 | 0.03943 | 0.002292 | 0.00971 | 0.02670 |
|  | K05569 | multicomponent Na+:H+ antiporter subunit E | 0.006322 | 0.000498 | 0.00949 | 0.001193 | 0.01079 | 0.0002 | 0.00728 | 0.02670 |
|  | K05570 | multicomponent Na+:H+ antiporter subunit F | 0.00262 | 0.000275 | 0.00451 | 0.000742 | 0.005034 | 0.000264 | 0.01832 | 0.02748 |
|  | K05571 | multicomponent Na+:H+ antiporter subunit G | 0.00392 | 0.000619 | 0.006069 | 0.000558 | 0.007183 | 0.000107 | 0.00728 | 0.02670 |
|  | K03315 | Na+:H+ antiporter, NhaC family | 0.005039 | 0.000728 | 0.007812 | 0.000518 | 0.008672 | 0.001038 | 0.01832 | 0.02748 |
| K+ transport | K03498 | trk system potassium uptake protein | 0.02798 | 0.002347 | 0.03439 | 0.002611 | 0.04126 | 0.002937 | 0.00971 | 0.02670 |
|  | K03499 | trk system potassium uptake protein | 0.04027 | 0.001255 | 0.04768 | 0.005637 | 0.06076 | 0.004706 | 0.01832 | 0.02748 |
|  | K01547 | potassium-transporting ATPase ATP-binding subunit | 0.03153 | 0.002236 | 0.03514 | 0.001916 | 0.02156 | 0.001978 | 0.00728 | 0.02670 |
|  | K01548 | potassium-transporting ATPase KdpC subunit | 0.008693 | 0.001133 | 0.01031 | 0.000761 | 0.005742 | 0.001247 | 0.01247 | 0.02725 |
|  | K11745 | glutathione-regulated potassium-efflux system ancillary protein KefC | 0.01044 | 0.000901 | 0.01042 | 0.000844 | 0.00554 | 0.000642 | 0.02491 | 0.02835 |
|  | K11746 | glutathione-regulated potassium-efflux system ancillary protein KefF | 0.001905 | 8.3e-05 | 0.001535 | 0.000715 | 0.000642 | 0.000329 | 0.02639 | 0.02903 |
|  | K11747 | glutathione-regulated potassium-efflux system protein KefB | 0.04526 | 0.002849 | 0.0404 | 0.00303 | 0.02845 | 0.004007 | 0.00971 | 0.02670 |

Note: Values are presented as the mean relative abundance ± standard deviation (SD) of three biological replicates. Selected KEGG orthologs (KOs) were grouped according to their annotated roles in compatible-solute synthesis, compatible-solute transport, Na⁺/H⁺ antiport, and K⁺ transport. Non-Sal, non-salinity treatment; Mid-Sal, moderate-salinity treatment; Hea-Sal, heavy-salinity treatment. P values indicate the overall differences in KO relative abundance among the three treatments. p< 0.05 , q < 0.05 was considered statistically significan.
